# Determining the cut-off score for the Modified Barthel Index and the Modified Rankin Scale for assessment of functional independence and residual disability after stroke

**DOI:** 10.1371/journal.pone.0226324

**Published:** 2020-01-29

**Authors:** Seung Yeol Lee, Deog Young Kim, Min Kyun Sohn, Jongmin Lee, Sam-Gyu Lee, Yong-Il Shin, Soo-Yeon Kim, Gyung-Jae Oh, Young Hoon Lee, Yang-Soo Lee, Min Cheol Joo, So Young Lee, Jeonghoon Ahn, Won Hyuk Chang, Ji Yoo Choi, Sung Hyun Kang, Il Yoel Kim, Junhee Han, Yun-Hee Kim

**Affiliations:** 1 Department of Physical and Rehabilitation Medicine, Center for Prevention and Rehabilitation, Heart Vascular and Stroke Institute, Samsung Medical Center, Sungkyunkwan University School of Medicine, Seoul, Republic of Korea; 2 Department and Research Institute of Rehabilitation Medicine, Yonsei University College of Medicine, Seoul, Republic of Korea; 3 Department of Rehabilitation Medicine, School of Medicine, Chungnam National University, Daejeon, Republic of Korea; 4 Department of Rehabilitation Medicine, Konkuk University School of Medicine, Seoul, Republic of Korea; 5 Department of Physical and Rehabilitation Medicine, Chonnam National University Medical School, Gwangju, Republic of Korea; 6 Department of Rehabilitation Medicine, Pusan National University Yangsan Hospital, Yangsan, Republic of Korea; 7 Department of Preventive Medicine, Wonkwang University School of Medicine, Iksan, Republic of Korea; 8 Department of Rehabilitation Medicine, Kyungpook National University, School of Medicine, Kyungpook National University Hospital, Daegu, Republic of Korea; 9 Department of Rehabilitation Medicine, Wonkwang University School of Medicine, Iksan, Republic of Korea; 10 Department of Rehabilitation Medicine, Jeju National University Hospital, University of Jeju College of Medicine, Jeju, Republic of Korea; 11 Department of Health Convergence, Ewha Womans University, Seoul, Republic of Korea; 12 Division of Chronic Disease Prevention, Korea Center for Disease Control and Prevention, Osong, Republic of Korea; 13 Department of Statistics and Institute of Statistics, Hallym University, Chuncheon, Republic of Korea; 14 Departmen of Health Sciences and Technology, Department of Medical Device Management & Research, Department of Digital Health, SAIHST, Sungkyunkwan University, Seoul, Republic of Korea; Massachusetts General Hospital, UNITED STATES

## Abstract

Assessment of functional independence and residual disability is very important for measuring treatment outcome after stroke. The modified Rankin Scale (mRS) and the modified Barthel Index (MBI) are commonly used scales to measure disability or dependence in activities of daily living (ADL) of stroke survivors. Lack of consensus regarding MBI score categories has caused confusion in interpreting stroke outcomes. The purpose of this study was to identify the optimal corresponding MBI and modified Rankin scale (mRS) grades for categorization of MBI. The Korean versions of the MBI (K-MBI) and mRS were collected from 5,759 stroke patients at 3 months after onset of stroke. The sensitivity and specificity were calculated at K-MBI score cutoffs for each mRS grade to obtain optimally corresponding K-MBI scores and mRS grades. We also plotted receiver operating characteristic (ROC) curves of sensitivity and specificity and determined the area under the curve (AUC). The K-MBI cutoff points with the highest sum of sensitivity and specificity were 100 (sensitivity 0.940; specificity 0.612), 98 (sensitivity 0.904; specificity 0.838), 94 (sensitivity 0.885; specificity 0.937), 78 (sensitivity 0.946; specificity, 0.973), and 55 (sensitivity 937; specificity 0.986) for mRS grades 0, 1, 2, 3, and 4, respectively. From this result, the K-MBI cutoff score range for each mRS grade can be obtained. For mRS grade 0, the K-MBI cutoff score is 100, indicating no associated score range. For mRS grades 1, 2, 3, 4, and 5, the K-MBI score ranges is from 99 to 98, 97 to 94, 93 to 78, 77 to 55, and under 54, respectively.The AUC for the ROC curve was 0.791 for mRS grade 0, 0.919 for mRS grade 1, 0.970 for mRS grade 2, 0.0 for mRS grade 3, and 0.991 for mRS grade 4. The K-MBI cutoff score ranges for representing mRS grades were variable; mRS grades 0, 1, and 2 had narrow K-MBI score ranges, while mRS grades 3, 4, and 5 exhibited broad K-MBI score ranges. mRS grade seemed to sensitively differentiate mild residual disability of stroke survivors, whereas K-MBI provided more specific information of the functional status of stroke survivors with moderate to severe residual impairment.

## Introduction

Stroke is a major global cause of serious, long-term disability and functional dependency [1]. Therefore, reducing the disability caused by stroke and improving the independence of stroke survivors are the primary goals of post-stroke rehabilitation [2]. Activities in daily living (ADL) are assessed as a measure of functional independency and outcomes of the rehabilitation process. Although numerous instruments are available for measuring disability and dependency in stroke survivors, the Barthel Index (BI) and the modified Rankin Scale (mRS) are the most widely used assessment tools in the clinic and in research [3].

The original version of the Barthel Index has 10 items, each scored in three steps [4]. The values assigned to each item are based on the amount of physical assistance required to perform the task. The Modified Barthel Index (MBI) with a five-step scoring system was developed by Shah et al.[5) and has greater sensitivity and reliability compared to the original version. The MBI and its translated versions in various languages provide a reliable measure of basic ADL for evaluating the effectiveness of rehabilitation. Therefore, it has been used frequently in large-scale outcome studies[6–10]. Although validated and used globally, the MBI has limitations in application and interpretation. While the MBI provides wide ranges of scores for ADL function, results interpretation is limited to numeric changes in total score. It is unclear that how much of the change in total score is clinically significant. Defining clinically distinct grades of ADL in stroke patients is an important issue not only for evaluating individual patient outcomes but also for evaluating population-level outcomes. However, there is no consensus about how many ADL levels could be meaningfully categorized in the MBI or the cutoff scores for those levels in each category.

In comparison, the mRS score is a clinically-based measurement of global disability using a 7-point scale ranging from 0 (no symptoms) to 6 (dead). While the mRS was originally designed as a handicap scale, it is now considered more of a disability scale [11]. mRS and MBI are often considered as similar measurements, and mRS has been widely applied to evaluate recovery from stroke and as a primary endpoint in clinical trials [12]. However, there are substantial difference between the two measurements. The crucial difference is that the mRS is a global disability scale, whereas MBI is a quantitative measurement of basic ADL. It has been argued that mRS consist of subjective categories and is difficult to reflect the degree of disabilities in detail. In fact, disability measured by mRS and the ADL measured by the MBI often revealed discrepancies in explaining the functional outcome of the stroke survivors [13]. Thus understanding the relationship between the mRS and MBI may help clinicians for interpreting treatment outcomes and designing clinical trial using both measure.

The aim of this study was to identify the optimal cutoff values for MBI scores to differentiate clinically distinct grades in ADL, thus revealing the correspondence between the mRS grade and MBI score and the transferability of these two measurements in assessing residual functional status of stroke survivors.

## Materials and methods

### Data collection

Data were obtained from the Korean Stroke Cohort for Functioning and Rehabilitation (KOSCO), a cohort of acute, first-ever stroke patients who were admitted to participating hospitals in 9 distinct areas of Korea [14, 15]. The KOSCO study was designed as a 10-year, longitudinal-follow-up study of stroke patients. It is a prospective multi-center project that investigates the residual disabilities, activity limitations, and long-term quality of life in patients suffering from first-time stroke. All eligible patients were recruited from August 2012 until May 2015. Patients formally entered the study after they provided written informed consent. If the patient was unable to make decision upon informed consent, it was obtained from the patient’s legally authorized representative. The study protocol was approved by Samsung Medical Center Institutional Review Board (approval number 2012-06-016). The KOSCO study included 7,858 first-ever stroke patients (6,254 ischemic and 1,604 hemorrhagic). Three months after stroke onset, 5,759 patients had completed the face-to-face follow-up assessment.

The MBI developed by Shah et al.[5] is a 100-point rating scale of a patient’s ability to perform 10 kinds of ADL. Each activity is assigned a numeric value according to the patient’s requirement for assistance. Lower scores indicate less independence, whereas higher scores indicate greater independence. Therefore, the maximum score of 100 represents a patient fully independent in performing basic ADL, whereas the lowest score (0) represents a totally dependent state. The MBI was translated into the Korean (K-MBI) by 6 Korean physiatrist experts in stroke. The contents of the test items were revised to reflect the Korean culture and lifestyle, and its validity and reliability were previously verified.(9) The mRS defines 6 different grades of disability and 1 for death [16]. mRS grade 6 was not included in this analysis because the present study focuses on the relationship between K-MBI and mRS, and patients with mRS grade 6 disability were unavailable to undergo K-MBI assessment.

K-MBI and mRS were obtained by face-to-face evaluator interviews with patients. To maintain optimum validity and interrater reliability, all assessments were performed by qualified evaluators who were licensed occupational or physical therapists and completed the standard training program provided by the KOSCO study. The standardized training program was held at the beginning of and every 3 months during the study period. The initial training program with a one-day workshop was performed three times before enrollment. The evaluators passed the standardized examination for functional assessments including on-line tests of the K-MBI and mRS to be approved to participate in data collection. The training program consisted of a one-day workshop and one-on-one education by an experienced evaluator for one week. The same regulation applied to additional evaluators who took part in the data collection.

### Analysis methods

All supporting data are available within the article. Statistical analyses were performed using the software package SPSS 24.0 (SPSS Inc., Chicago, IL, USA). Because the data do not satisfy the assumption of normal distribution, all statistical tests applied were nonparametric. Descriptive statistics were used to examine the distribution of the K-MBI scores according to mRS grade. The Chi-square test was performed to find the significance in proportion of sex among mRS grades The Kruskal-Wallis test was applied to examine the mean difference in age and the K-MBI scores among mRS grades. Mann-Whitney tests were used to examine which pairs were significantly different.

The sensitivity, specificity, and positive and negative predictive values (PPV and NPV) of the K-MBI scores for each mRS grade were calculated at all possible cutoff points. The mRS grades were dichotomized into two categories. For example, to calculate corresponding K-MBI scores for mRS grade 1, mRS 0 or 1 was set as “favorable outcomes,” and mRS 2 to 4 were set as “unfavorable outcomes.” Sensitivity refers to the proportion of cases that were above or equal to the K-MBI cutoff score among the favorable outcomes. Specificity refers to the proportion of cases that were below the K-MBI cutoff scores among the unfavorable outcomes. The PPV is the proportion of favorable outcomes according to mRS among the cases above the K-MBI cutoff score. The NPV is the proportion of unfavorable outcomes among the cases that are below the K-MBI cutoff score. The optimal cutoff score was determined as the score with the highest sum of sensitivity and specificity [17].

Receiver operating characteristic (ROC) analysis was performed, and the area under the curve (AUC) was calculated to investigate the relationship between sensitivity and specificity. ROC curves plot sensitivity versus 1-specificity, enabling visualization of the optimal cutoff K-MBI score for each mRS grade. The AUC indicates the discrimination potential of the K-MBI cutoff score in the mRS grade, with a higher AUC reflecting better performance [18]. A permutation test was performed to evaluate the significance of an AUC value.

## Results

### Population characteristics

**Fig 1:**
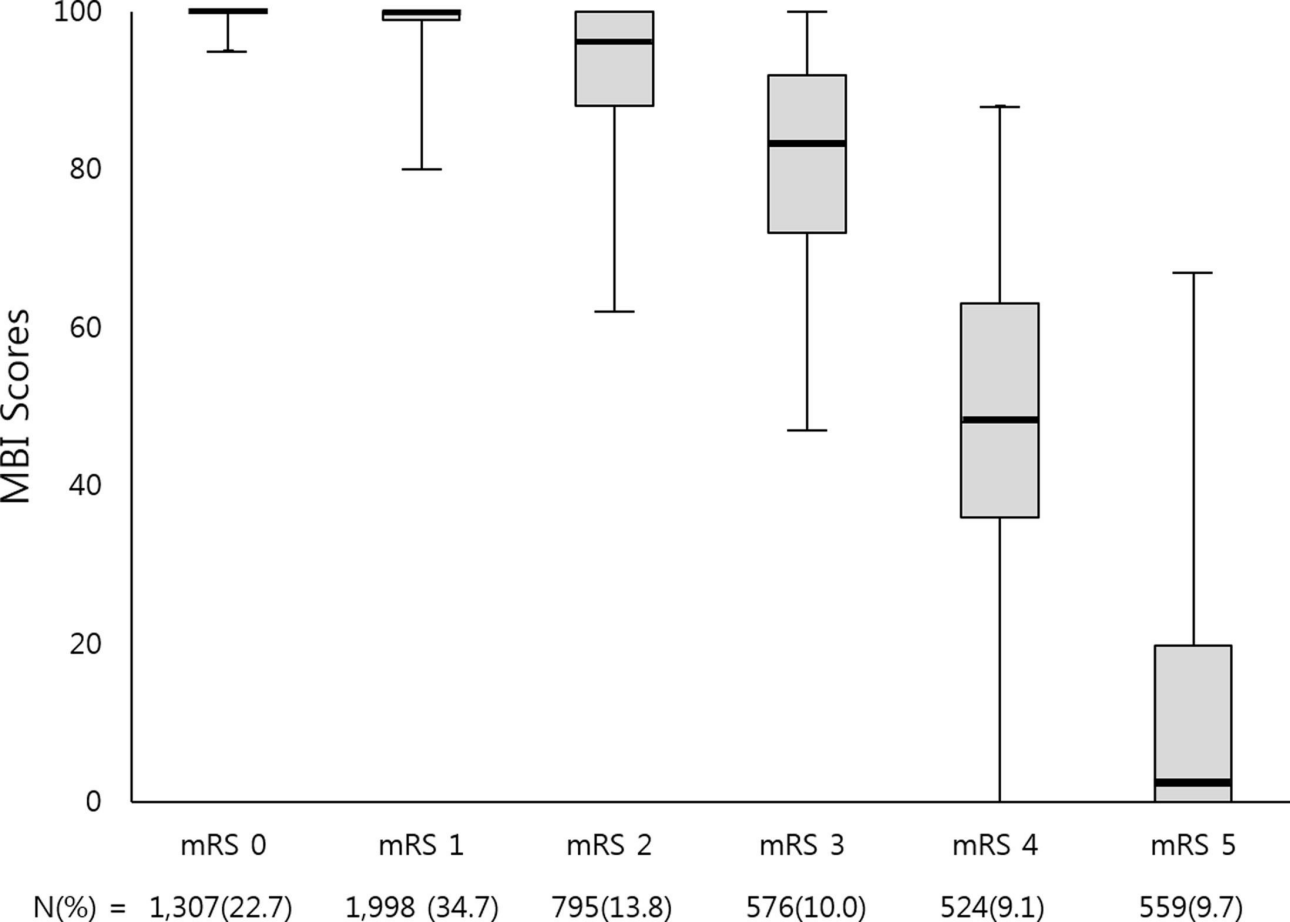
Distribution of K-MBI scores within the mRS grades. The central rectangle spans the first to the third quartiles. The segment inside the rectangle shows the median and whiskers above and below the box show the locations of the maximum and minimum, respectively. K-MBI, Korean version of Modified Barthel Index; mRS, modified Rankin Scale.

Of the 5,759 patients, 3,332 (57.9%) were male. The mean age was 64.1 years (SD 13.2 years). The median K-MBI score was 65, with an interquartile range from 55 to 74. Fig 1 illustrates the K-MBI frequency distribution in each MRS grade and demonstrates the median, interquartile range, minimum, and maximum of the K-MBI scores. The mRS score distribution was as shown in Table 1.

**Table 1:** Numbers and demographic characteristics of mRS grades.

	Number (%)	No. of Male (%)	Age (Mean±SD)
mRS Grade 0	1304(22.7)	870(66.7)	58.92±12.94
mRS Grade 1	1997(34.7)	1212(60.7)	62.67±12.99
mRS Grade 2	796(13.8)	431(54.2)	65.20±12.23
mRS Grade 3	577(10.0)	316(54.9)	65.85±12.93
mRS Grade 4	525(9.1)	250(47.6)	68.59±12.72
mRS Grade 5	559(9.7)	253(45.3)	72.99±11.34

mRS, modified Rankin Scale; SD, Standard deviation

The proportion of sex and mean age among mRS grades were significantly different (*p*<0.05). In post hoc analysis, proportion of sex in mRS grades were significantly different to each other, except for mRS grade 2 and mRS grade 3, and for mRS grade 4 and mRS grade 5. Mean age of mRS grades was significantly different to each other, except for mRS grade 2 and mRS grade 3.

The range of K-MBI scores was small in the mRS 0, 1, and 2 categories, and the medians were similar (100, 100, and 96, respectively). In contrast, the range of K-MBI scores in the mRS 3, 4, and 5 categories was wide, with median scores of 83, 48, and 2, respectively. There was considerable overlap in K-MBI scores between mRS grades.

Kruskal-Wallis tests were applied to determine whether there was a statistically significant difference (*P*<0.05) in the mean K-MBI scores among mRS grades. The results showed that there were significant differences in the K-MBI scores among mRS grades (*P*<0.0001). The Mann-Whitney test was performed as a post-hoc analysis of the Kruskal-Wallis test. All mRS grades were statistically distinct from one another (*P*<0.01).

### The optimal K-MBI cutoff scores for mRS grades

The optimal K-MBI cutoff scores in relation to mRS grades and the sensitivity, specificity, PPV, and NPV of each cutoff are shown in Table 2.

**Table 2:** Sensitivity, specificity, sum score, PPV, and NPV for K-MBI cutoff scores corresponding to mRS grades.

	K-MBI Cutoff Score	Sensitivity	Specificity	Sum Score*	PPV	NPV
mRS Grade 0	100	0.940	0.612	1.552	0.413	0.972
mRS Grade 1	≥98	0.904	0.838	1.742	0.881	0.868
mRS Grade 2	≥94	0.885	0.937	1.822	0.971	0.769
mRS Grade 3	≥78	0.946	0.973	1.919	0.993	0.810
mRS Grade 4	≥55	0.937	0.986	1.923	0.999	0.621

*Sum Score = Sensitivity + Specificity

PPV, Positive predictive value; NPV, Negative predictive value; K-MBI, Korean version of Modified Barthel Index; mRS, modified Rankin Scale; SD, Standard deviation

For mRS grade 0, the optimal K-MBI cutoff score was 100, with a sensitivity of 0.940 and a specificity of 0.612. For mRS grade 1, the optimal K-MBI cutoff score was 98, with a sensitivity of 0.904 and a specificity of 0.838. For mRS grade 2, the optimal K-MBI cutoff score was 94, with a sensitivity of 0.885 and a specificity of 0.937. For mRS grade 3, the optimal K-MBI cutoff score was 78, with a sensitivity of 0.946 and a specificity of 0.973. For mRS grade 4, the optimal K-MBI cutoff score was 55, with a sensitivity of 0.937 and a specificity of 0.986. From this result, the K-MBI cutoff score range for each mRS grade was obtained. For mRS grade 0, the K-MBI cutoff score was 100, indicating no associated score range. For mRS grades 1, 2, 3, 4, and 5 the K-MBI score range was from 99 to 98, 97 to 94, 93 to 78, 77 to 55, and under 54, respectively.

The AUC for the K-MBI cutoff score was 0.791 in mRS grade 0, 0.919 in mRS grade 1, 0.970in mRS grade 2, 0.994) in mRS grade 3, and 0.991 in mRS grade 4 (Table 3).

**Table 3:** Area under the curve (AUC) of the K-MBI cutoff scores corresponding to mRS grades.

	Area	SE	Asymptotic Significance	Asymptotic 95% Confidence Interval	
				Lower Bound	Upper Bound
mRS Grade 0	0.791	0.004	<0.0001	0.780	0.802
mRS Grade 1	0.919	0.004	<0.0001	0.912	0.926
mRS Grade 2	0.970	0.002	<0.0001	0.965	0.974
mRS Grade 3	0.994	0.001	<0.0001	0.991	0.996
mRS Grade 4	0.991	0.001	<0.0001	0.988	0.993

K-MBI, Korean version of Modified Barthel Index; mRS, modified Rankin Scale; SE, standard error

**Fig 2:**
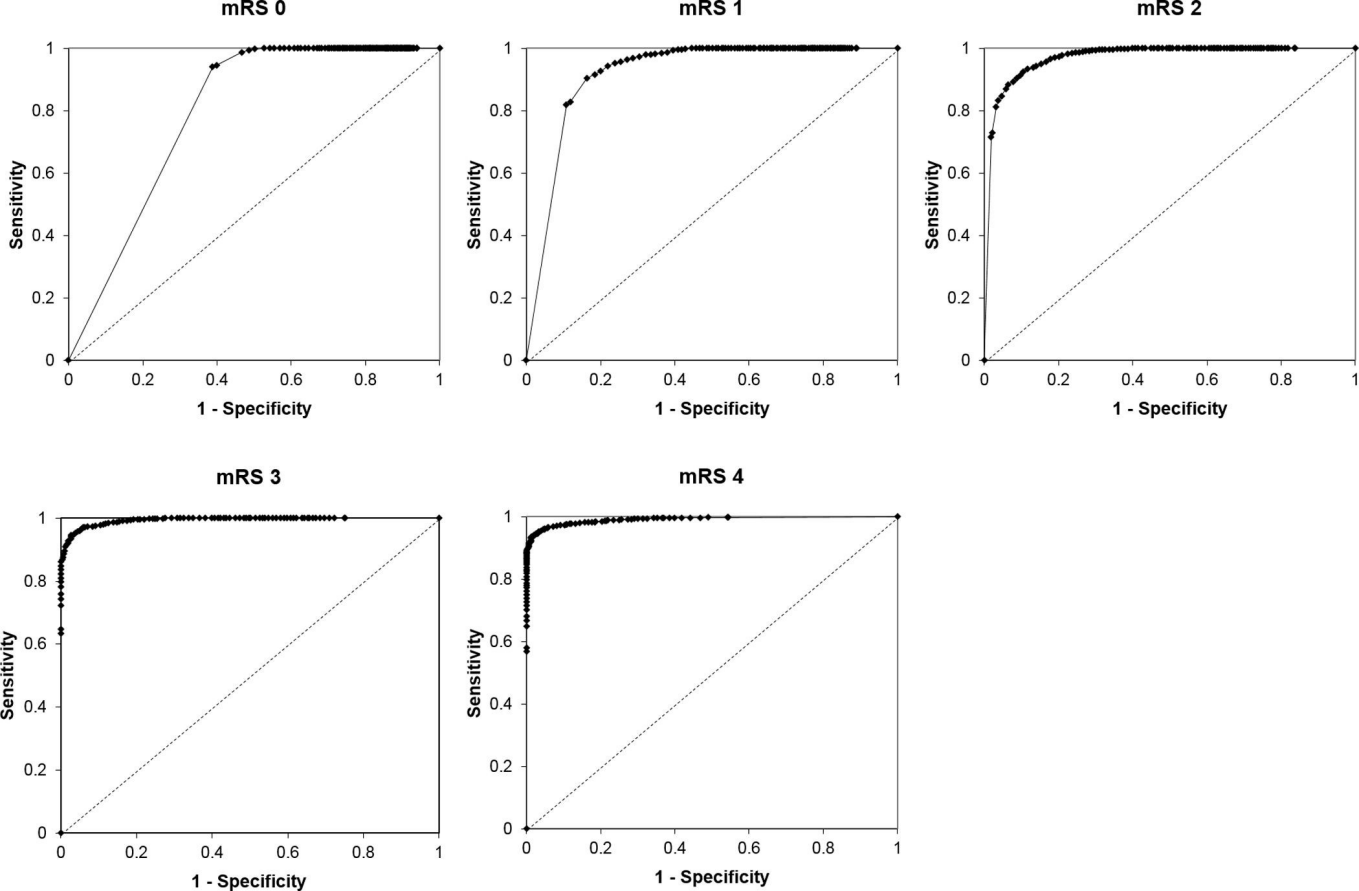
ROC curves of K-MBI cutoff scores corresponding to mRS grades. ROC, Receiver operating characteristic; K-MBI, Korean version of Modified Barthel Index; mRS, modified Rankin Scale.

In all mRS grades, asymptotic significance for all AUC were all very significant (p < 0.0001), which indicates that all K-MBI cut-off scores are suitable measurement for discrimination of dichotomized mRS grades (AUC larger than 50%). Fig 2 shows the ROC curves.

## Discussion

In this study, we analyzed the correspondence between mRS and K-MBI by calculating optimal K-MBI cutoff scores for mRS. The K-MBI cutoff scores were determined as 100 for mRS grade 0, 98 for mRS grade 1, 94 for mRS grade 2, 78 for mRS grade 3, and 55 for mRS grade 4. To our knowledge, this is the first study to determine the K-MBI cutoff scores corresponding to the mRS grades in stroke patients. K-MBI enables evaluation of the patient’s basic ADL from multiple perspectives and is being increasingly adopted as a primary endpoint in clinical trials of stroke[14, 19–22]. The results from this study may have consequences for determining outcomes in stroke trials. For example, a change in K-MBI category suggests clinically meaningful improvement of ADL function in stroke patients. Therefore, researchers can decide whether the change in score within a group indicates intervention benefit. In addition, it is also possible to dichotomize the outcomes of stroke trials as favorable or unfavorable. Although there is no agreed upon dichotomization point for mRS[23]. most stroke trials define a favorable outcome as mRS grade ≤ 1 or ≤ 2([24, 25]. According to our results, if trials define a favorable outcome, the target score after intervention, or recovery time is mRS grade ≤ 1 with a corresponding K-MBI cutoff score ≥ 98. If trials define a favorable outcome as mRS grade ≤ 2, the corresponding K-MBI cutoff score would be ≥ 94. Moreover, translation of outcomes across trials may enable meta-analyses in acute stroke trials.

An interesting finding from this study is that the K-MBI cutoff score ranges representing mRS grades were variable. In particular, mRS grades 0, 1, and 2 had a narrow range for K-MBI scores. Although the mean K-MBI values for mRS grades 0, 1, and 2 were significantly different, the differences between the optimal K-MBI cutoff scores for mRS grade 0 and mRS grade 1 and for mRS grade 1 and mRS grade 2 were 2 points and 4 points, respectively. These K-MBI cutoff scores are near the maximum, which indicates a ceiling effect of K-MBI. Although a ceiling effect has not been previously reported for K-MBI, such an effect is frequently observed for BI [26, 27]. Since K-MBI is based on and highly correlated with BI, it would not be surprising if the ceiling effect was also observed in K-MBI. The K-MBI focuses on basic ADL and lacks information on many instrumental ADLs. Therefore, many tasks that are not measured with K-MBI could play an important role in disability after stroke. A K-MBI score of 100 (independent on all 10 activities) does not mean that a patient is able to carry out advanced ADLs independently. These findings indicate that K-MBI may not be a sensitive evaluation tool in detecting changes in disabilities in mild stroke patients.

In contrast, mRS grades 3, 4, and 5 exhibited a broad range of K-MBI scores, and the K-MBI scores overlapped between the mRS grades. This may be due to the poor representative value of mRS in patients with moderate to severe disabilities, suggesting that that mRS is not a sensitive measure for patients in these categories. Rather, the K-MBI might have higher discrimination capability in patients who have moderate to severe disabilities. These findings are consistent with previous research. In stroke patients with mild to moderate disability, mRS has better responsiveness since BI experiences ceiling effect. However, in patients with severe disability, BI was more useful for discriminating patients [28]. In the same vein, global scales, such as mRS, were much less sensitive to changes in disability than were ADL scales [29].

The mRS is very simple and requires no special tools or training, which makes it feasible for use in large trials. Although the intra-rater reliability of mRS is relatively satisfactory, inter-rater reliability was found to be low, particularly in studies with larger sample size [30]. This may be due to the subjective nature of the score and lack of clear criteria, which reduced the reliability of the measurements [31–33]. Furthermore, a 7-point ordinal scale may be insufficient to precisely evaluate patient disability. mRS grades are determined by the limitations in activities and mobility of patients. However, limitation in daily activities and mobility of stroke patients do not always present in typical stages. There have been attempts to improve the reliability of mRS. Previous studies has reported that structured interview can increase inter-rater reliability [31, 32) and even suggested that the use of the ADL checklist for scoring of mRS could improve inter-rater reliability [34].

In this study, we used the mRS as a reference to categorize K-MBI. The primary objective of this study was to suggest clinically distinguishable K-MBI categories with statistical evidence. Although mRS is not the gold standard for disability measurement in stroke patients, we believe it is suitable for this purpose. First, mRS is the most popular assessment of global disability in stroke with well-studied validity and reliability. Second, 6 levels of mRS are well-defined and clinically distinct.

This study has a few limitations. First, a total of 2,099 (26.7%) patients missed the 3 month follow up visit after stroke onset. Exclusion of these patients may have had some influence on the results. However, the objective of this study was to determine the cutoff scores in K-MBI for mRS. For this purpose, a large number of K-MBI and mRS scores evaluated at the same time point from the same patient are required. In the KOSCO study K-MBI and mRS were evaluated at the initial time point, at 7 days after stroke, and followed up at 3 month at the same time in the same patients. Although a substantial number of patients were lost at the 3 month follow-up, the data from this period suit the aim of this study. Second, in this study, the results represent the whole sample size. The results were not stratified for gender, age-categories, co-morbidities and different time points (at baseline and after a few months). Despite the established reliability of mRS and K-MBI, the results obtained may not be generalizable to other gender or age group. In addition, the study used K-MBI in an exclusively Korean population. Further studies should explore the validity of cut-off scores from this study across different population.

## Conclusions

Overall, even though mRS and K-MBI are often categorized as functional outcome scale in stroke trials, they have substantially different characteristics. Neither mRS nor K-MBI offers a complete picture of functional ability in stroke patients. mRS seems to be more sensitive than K-MBI in stroke survivors with milder residual disability, whereas K-MBI can be more sensitive in stroke survivors with severe residual disability by providing more specific information about ADLs. Therefore, careful attention must be given when using of one these measurements in assessing residual disability of stroke survivors.

## Abbreviations

ADL: Activities in daily living
MBI: Modified Barthel Index
mRS: Modified Rankin Scale
K-MBI: Korean version of the Modified Barthel Index
PPV: Positive predictive values
NPV: Negative predictive values
ROC: Receiver operating characteristics
AUC: Area under the curve
